# Detector clothes for MRI: A wearable array receiver based on liquid metal in elastic tubes

**DOI:** 10.1038/s41598-020-65634-5

**Published:** 2020-06-01

**Authors:** Andreas Port, Roger Luechinger, Loris Albisetti, Matija Varga, Josip Marjanovic, Jonas Reber, David Otto Brunner, Klaas Paul Pruessmann

**Affiliations:** 1 Institute for Biomedical Engineering, ETH Zurich and University of Zurich, Zurich, Switzerland; 2 Institute for Electronics, ETH Zurich, Zurich, Switzerland

**Keywords:** Magnetic resonance imaging, Biomedical engineering, Electrical and electronic engineering

## Abstract

In modern magnetic resonance imaging, signal detection is performed by dense arrays of radiofrequency resonators. Tight-fitting arrays boost the sensitivity and speed of imaging. However, current devices are rigid and cage-like at the expense of patient comfort. They also constrain posture, limiting the examination of joints. For better ergonomics and versatility, detectors should be flexible, adapt to individual anatomy, and follow posture. Towards this goal, the present work proposes a novel design based on resonators formed by liquid metal in polymer tubes. Textile integration creates lightweight, elastic devices that are worn like pieces of clothing. A liquid-metal array tailored to the human knee is shown to deliver competitive image quality while self-adapting to individual anatomy and adding the ability to image flexion of the joint. Relative to other options for stretchable conductors, liquid metal in elastic tubes stands out by reconciling excellent electrical and mechanical properties with ease of manufacturing.

## Introduction

Magnetic resonance imaging (MRI) is an indispensable tool to obtain anatomical and functional information from inside the living organism. With its excellent soft tissue contrast, high spatial resolution and no ionizing radiation, it has found widespread use in today’s clinics and research.

MRI relies on the combined effects of three magnetic fields. A several Tesla strong static field is commonly generated by a superconducting magnet. Radiofrequency and gradient fields are employed for excitation of tissue and spatial encoding, respectively. Precise actuation of the latter two fields through MRI sequences gives rise to the generation of MR signals from which tomographic images are calculated. Emitted MR signals are small in amplitude and are captured by highly sensitive receive coils.

The nature of receive coils has changed considerably over time making MRI more sensitive and widely applicable. Single channel receive coils were used in the beginnings of MRI, but a major paradigm shift took place in the 1990s and early 2000s with the emergence of multiple channel receiver coil arrays^1^ and parallel imaging^2–4^. Higher signal-to-noise ratio (SNR) and shorter scans were the result.

**Figure 1:**
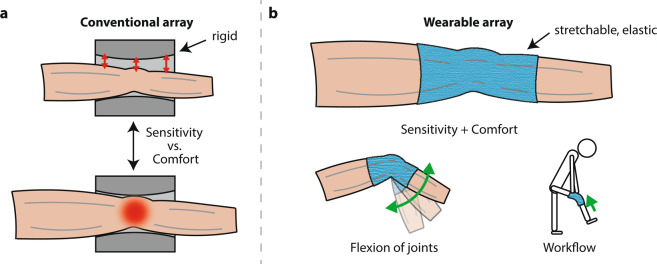
Conventional and wearable MR detection. **(a)** A conventional coil array is rigid and does not adapt to the patient’s anatomy. Distance between body part and coil array compromises sensitivity. Conversely, for some patients, comfort is limited by tightness of the array, reflecting a tradeoff that is inevitable with a device of fixed size and shape. **(b)** A wearable array is stretchable and elastic. It adapts in size and shape to the patient’s anatomy, achieving high sensitivity and patient comfort at the same time. A wearable array also allows for flexion of joints and promises enhanced workflow as it can be put on by patients themselves.

Conventional receive coil arrays used in current clinical practice are rigid and of fixed size as exemplified for knee imaging in Fig. 1a. While for improved sensitivity it is desired that the coil array closely surrounds the anatomy of interest, the array must also fit a large variety of anatomical sizes. The resulting size of a conventional coil array thus inevitably limits attainable sensitivity in some patients and comfort in others.

Today, receive coil arrays are undergoing another major paradigm shift, which aims to overcome this limitation by increased adaptability to the patient’s anatomy. To achieve this adaptability, alternatives must be sought to current coil array implementations, which commonly employ inflexible coil elements made from rigid copper for signal reception.

While this goal has been partly reached with rigid-adjustable^5–9^ and flexible^10–19^ coil designs, full adaptability arguably requires that coils also be stretchable. One stretchable design is based on braided copper^20^, which provides high conductivity but requires textile substrates with large, reproducible and carefully balanced restoring forces. Another approach uses conductive thread^21^ sewn onto a stretchable textile. While providing simple manufacturing, use of thin yarn limits electrical performance unless deployed in large amounts, which again comes with mechanical challenges. In another work, a stretchable conductor is formed by stencil-printing liquid Gallium Indium metal onto neoprene foam^22,23^. This approach requires a non-permeable substrate with a porous surface structure for the liquid metal to adhere to. Due to limited thickness of printed liquid-metal layers, somewhat lower electrical performance has been achieved so far. Challenges in terms of sealing and contacting of the liquid metal phase remain.

Against this background, the quest continues for highly stretchable conductors with excellent electrical and mechanical performance.

In this work, we demonstrate an alternative liquid-metal implementation that reconciles high electrical performance with ease of manufacturing, simple sealing and large stretching range. Eutectic Gallium Indium liquid metal is contained in polymer tubes as previously done for forming flexible conductors^10,11^. Highly stretchable, thin silicone tubing is used with suitable volume-strain characteristic for containing the liquid metal under sealed conditions. Highly stretchable MR coils are formed that fully satisfy requirements concerning sealing and electrical conductance. Combining these coils into an adaptive, wearable receive array (Fig. 1b) allows for static and kinematic MR imaging of the human knee. The wearable array not only offers high sensitivity along with patient comfort, but also enables MR imaging of the flexion of the joint. In addition, it promises workflow enhancements as the wearable array can be put on by patients themselves, which could reduce setup times.

## Results

### Components and fabrication of the liquid metal coils

Eutectic Gallium Indium (eGaIn) is liquid at temperatures above 15.7 °C/60.3 °F. It possesses low vapor pressure and low toxicity^24^. Its resistivity^25^ is ${\rho }_{eGaIn}=29.4\,\cdot \,{10}^{-8}\,\Omega m$. At DC, this is 17 times larger than the resistivity of copper, ${\rho }_{Cu}=1.68\,\cdot \,{10}^{-8}\,\Omega m$. At radiofrequencies (RF), however, the effective cross section of a conductor carrying current is reduced by the skin effect. The skin depth is 24 μm for eGaIn and 6 μm for copper at a Larmor frequency of 128 MHz for a 3T MRI system. Thus, the material with higher resistivity maintains a larger current cross section, partly compensating its drawbacks as a bulk conductor. For conductors significantly thicker than the skin depths, replacing copper by eGaIn increases the resistance at 128 MHz by only a factor of four^10^.

**Figure 2:**
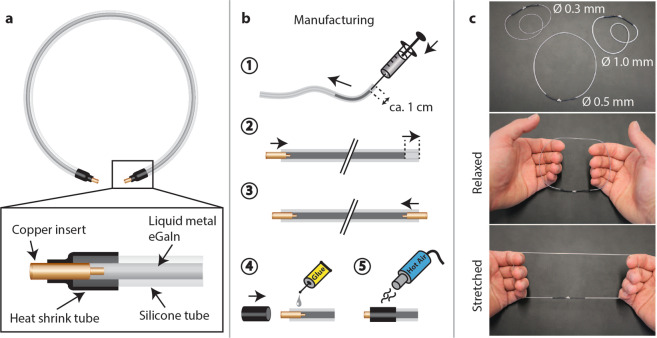
The liquid metal coils. **(a)** Liquid metal is contained in a stretchable silicone tube. A copper insert together with heat shrink tubing forms a sealed and solderable electrical contact to the liquid metal. **(b)** A syringe is used to inject the Gallium Indium liquid metal into the silicone tube of desired length. The needle with diameter fitted to the inner diameter of the silicone tube is inserted into the tube about 10 mm deep (Step 1). The copper contact is pushed into the opposite side of the tube causing the liquid metal to flow and empty the tube of remaining air (Step 2). A second copper contact is inserted on the remaining open side (Step 3). Instant adhesive and heat shrink tubing are applied to fully seal the contact (Steps 4 and 5). **(c)** Liquid metal coils of various inner diameters have been assembled and exhibit excellent stretchability.

The liquid metal is contained in silicone tubes (Fig. 2a), which are cut to the length of the desired coil dimensions. Tubes of different inner and outer diameters may be used. Their specified stretchability of more than 500% was verified by breakdown strain measurements. Silicone tube has a Poisson ratio of approximately 0.5. At this ratio, the silicone tube exhibits isovolumetric behavior under strain, which limits pressure changes and thus is favorable for containing a liquid under sealed conditions^26^.

Solderable electrical contact to the liquid metal is formed by either copper litz wire or a custom copper insert shaped to increase surface contact area. Copper is not subject to corrosion or oxidation with eGaIn^24^. Instant adhesive and heat shrink tubing are applied for sealing of the contact. The force resulting in breakdown of the contacts was evaluated to be more than 8 N.

The manufacturing process (Fig. 2b) aims to produce stretchable conductors with high conductance. No air enclosures can be tolerated inside the tube as they restrict current flow, which results in hampered electrical performance. The eGaIn is injected into the silicone tube using a syringe. Two copper contacts are inserted subsequently on both sides of the filled tube.

Resulting coils of different conductor diameters (Fig. 2c) provide high mechanical robustness and are highly stretchable.

### Liquid metal coil performance at different conductor diameters

**Figure 3:**
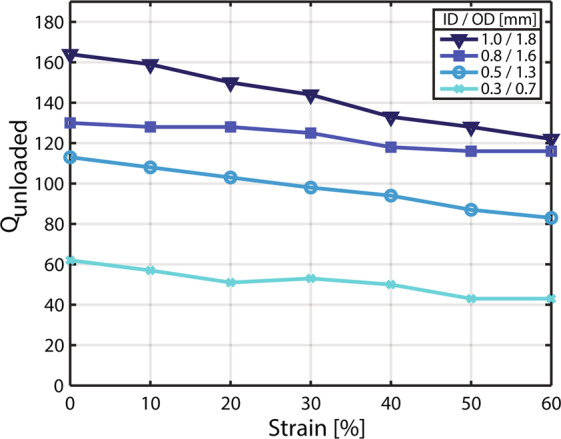
Unloaded quality factor measurements for liquid metal coils of different tube inner diameters. Q_unloaded_ is shown for coils stretched radially from 0 to 60% in 10% steps. Measurements are shown for coils with tube inner diameters (ID) of 0.3, 0.5, 0.8 and 1.0 mm. Respective outer diameters (OD) are 0.7, 1.3, 1.6 and 1.8 mm.

The unloaded quality factor Q_unloaded_, an indicator for the resistive losses of the coil, was measured while coils with various tube diameters were radially stretched by 0 to 60% (Fig. 3). With increasing tube inner diameter from 0.3 to 1.0 mm, Q_unloaded_ increased from 62 to 164 without applied strain. For strains of 60%, Q_unloaded_ decreased by 26 to 31% relative to the initial value without strain for tube inner diameters of 0.3, 0.5 and 1.0 mm. The 0.8 mm inner diameter tube coil showed a decrease of 11% when stretched by 60%. All coils show high initial Q_unloaded_, with only moderate decrease when stretched. Resonance frequency decreases and coil loss increases when the coils are stretched (Supplementary Fig. S1).

The loaded quality factor Q_loaded_ of a coil reflects not only losses of the coil itself but also sample losses. A Q ratio of Q_unloaded_/Q_loaded_ ≈ 10 measured for the 0.8 mm and 1.0 mm inner diameter tube coils indicates that coil losses are clearly dominated by sample losses. This is desired for SNR-efficient detection. A copper reference coil of the same dimensions exhibited Q_unloaded_/Q_loaded_ ≈ 15.

Using cyclic strain measurements, the behavior of Q_unloaded_ was evaluated over several hundred strain cycles. No degradation of Q_unloaded_ was measured and no mechanical failure occurred, indicating mechanical and electrical robustness. Rechecking Q_unloaded_ after several weeks revealed no degradation, suggesting that no performance limitations through corrosion or oxidation on interfaces between copper contacts and the liquid metal occurred.

**Figure 4:**
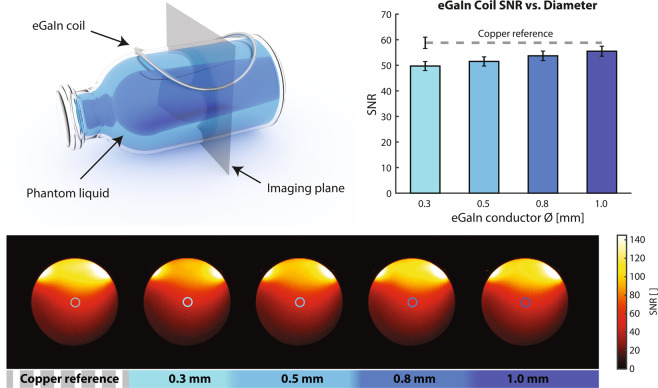
SNR comparison of liquid metal coils with various tube diameters. Coils are attached to a phantom bottle containing a copper sulfate solution emulating MR characteristics of human tissue. A transverse imaging plane was chosen for SNR evaluation. SNR data is plotted as mean SNR inside a circular region of interest in the center of the phantom. Error bars show standard deviation of this SNR data.

To evaluate the SNR performance of the liquid metal coils in comparison with a copper reference coil, MR imaging was performed in a phantom (Fig. 4 and Supplementary Fig. S2). SNR maps were derived from image and noise data^7^. The SNR yield of the stretchable coils, taken in the center of the phantom, approaches that of the copper reference as the tube inner diameter increases. The 0.8 mm coil achieved 91% of the SNR of the copper coil. The liquid metal coil with the smallest tested inner diameter of 0.3 mm and corresponding lower Q_unloaded_ still achieved 85% of the SNR of the copper reference coil, reflecting dominant loading by the phantom (Fig. 4).

### Multi-channel imaging

**Figure 5:**
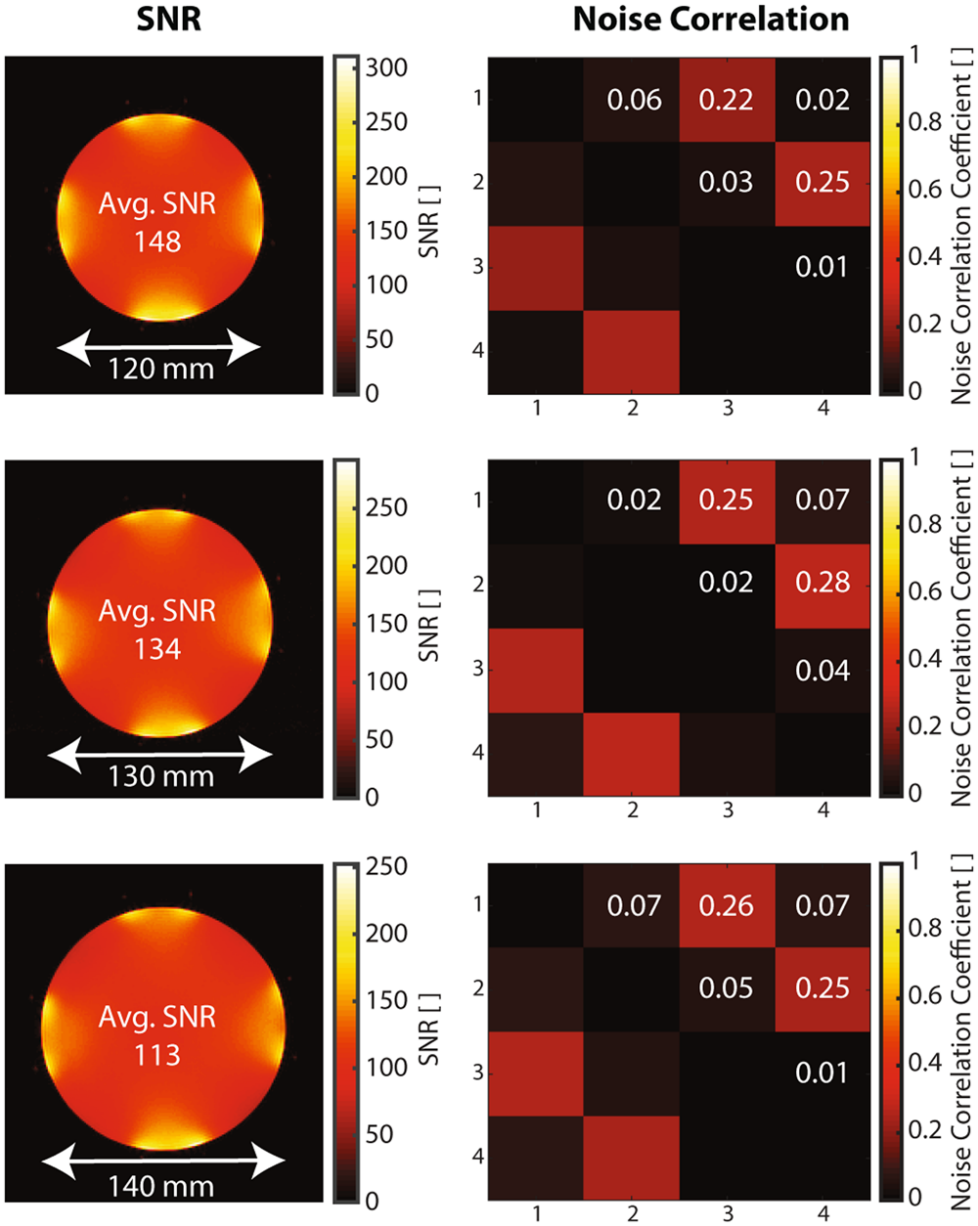
Coil array SNR performance when stretched around phantoms of increasing diameter. SNR maps, average SNR, and noise correlation maps are shown for phantoms of diameters 120 mm, 130 mm and 140 mm.

Receive coil arrays enable high-sensitivity imaging and are designed to provide coverage for a specific anatomy. For coverage of a typical adult knee size, four stretchable liquid metal coils were combined into a receive array. For phantom SNR evaluation, circular coils were sewn onto a stretchable cylindrical textile with mutual overlap for approximate geometric decoupling. Interface electronics provided preamplifer decoupling^1^. SNR maps derived from imaging of three differently sized phantoms (Fig. 5) show that uniform SNR distribution is maintained when the array is stretched. The average SNR undergoes some penalty at larger phantom sizes, which is expected from correspondingly lower sensitivity at larger distances from the coil elements and noise from a larger sample volume. Corresponding noise correlation maps indicate low inter-element coupling, which is well maintained when the coil array is stretched.

### The wearable knee array

**Figure 6:**
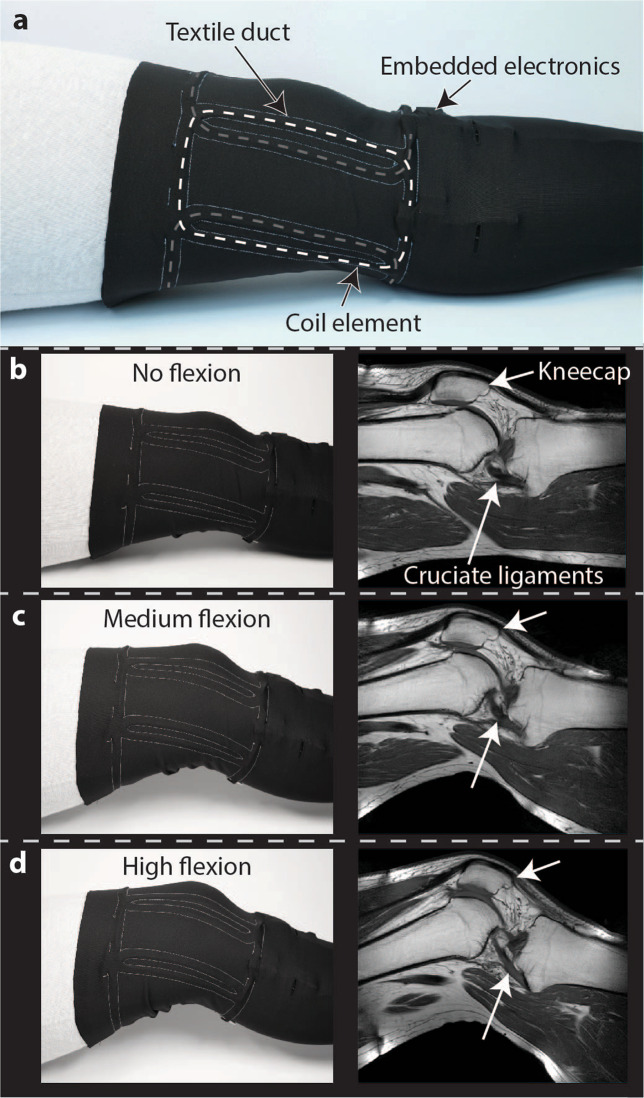
Wearable coil array used for *in vivo* imaging of the knee. **(a)** Receive coil array with textile-integrated liquid metal coils. Photographs and *in vivo* images of the knee in **(b)** straight **(c)** medium flexed and **(d)** highly flexed static position.

A stretchable textile substrate is used to form a wearable coil array. It should be thin, offer high elasticity in two dimensions, and provide comfortable fit. Given these requirements, commercially available athletic pants made from 79% polyester and 21% elastane have been found to be suitable. Two layers of this stretchable textile were connected by a sewing pattern that generates textile ducts into which four liquid metal coils are inserted (Fig. 6a). Individual coil element geometry and placement was chosen to create a wearable coil array that permits flexion of the knee joint. Each coil element was connected to custom, textile-embedded electronics for tuning, detuning, matching of the coil and signal preamplification. Between the unloaded state and mounting on a volunteer, all coil elements showed Q ratios of 11 or higher, indicating clear sample noise dominance.

*In vivo* images, employing a clinical RARE sequence, were acquired at three different flexion angles of a volunteer’s knee (Fig. 6b–d). These images confirm sensitivity and coverage of the wearable array. Sensitivity remains unchanged when the knee is flexed, indicating robust tuning and matching of the coil array. Decoupling of coil elements is well maintained upon flexion. Hyperintensities in skin areas indicate position of coil conductors as a characteristic of a close fitting surface coil, but do not limit radiological assessment. Movement of the kneecap relative to the thighbone and rearrangement of the cruciate ligaments upon flexion of the knee are observed.

**Figure 7:**
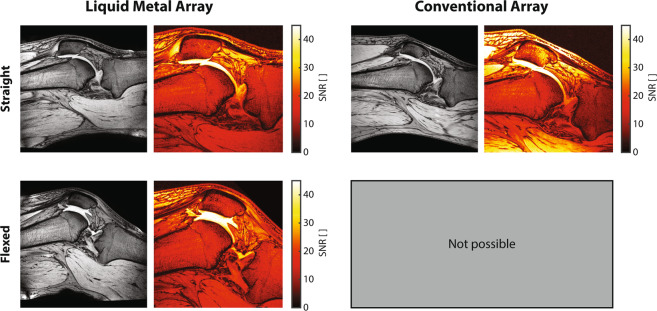
Comparison of the wearable liquid metal array with a conventional array. *In vivo* images and SNR maps of a region of interest around the center of a volunteer’s knee. Data for flexion of the joint shown for the wearable coil array; flexion is not possible in the conventional coil array.

Comparison between the 4-channel wearable and a widely used commercial 8-channel knee array was performed (Fig. 7). *In vivo* images demonstrate comparable visual impression between the wearable and the commercial knee array. Diagnostic utility is limited by the lowest SNR in the region of interest. Here, for both coil arrays this area is in the center of the knee and, with comparable SNR values. Higher SNR is achieved by the commercial array close to the surface, particularly in skin and muscle, due to a higher number of channels for approximately the same coverage. The SNR remains unaltered for the wearable array when the joint is flexed, confirming robust tuning and matching. Flexion of the knee is not possible in the commercial array.

Beyond static, fixed-angle imaging, the wearable array additionally enables kinematic imaging (Supplementary Vid. S1). Using a SPGR sequence, within 1:22 min 30 dynamics of a sagittal slice of a volunteer’s knee were acquired while the joint was continuously flexed in the MR scanner. High sensitivity and coverage are obtained over the whole range of flexion of the knee joint, indicating well controlled tuning, matching and decoupling of coil elements even during continuous flexion. Steady movement of the kneecap and cruciate ligaments can be observed.

## Discussion

Stretchable MR coils made from eutectic Gallium Indium liquid metal contained in silicone tubes are feasible. They offer mechanical and electrical robustness, providing high initial Q_unloaded_, with only a moderate decrease over a large range of strain. Notably, compared to other tube diameters, the 0.8 mm inner diameter tube coil showed somewhat less variation of Q_unloaded_ with strain. While this behavior is not fully understood yet, it is likely related to the characteristics and geometry of the silicone tubes and contacts. When coils are stretched, the tube cross section undergoes slight warping that depends on the tube diameter and relative wall thickness and may influence the resistive and inductive behavior of coils. Likewise, the contacts between liquid metal and litz wire may exhibit some sensitivity to strain-induced changes in the tube geometry, altering resistive behavior differently for different tube diameters.

Compared to a copper reference, the liquid-metal coils incurred only a minor SNR drawback as reported in Fig. 4. The underlying experiments focused on differences in SNR yield associated with the conductor materials and stretching. They did not aim to strictly maximize the SNR in absolute terms, e.g., by an advanced choice of preamplifier or by segmentation. Segmentation is a common means of optimizing sensitivity. However, it will alter the SNR comparison only to the degree to which voltages across the capacitors, and thus dielectric losses, differ between the copper reference coil and the liquid metal coils. Given very similar loaded Q of 18 for copper and 16 for liquid metal, the differences in these voltages were rather small, implying little difference also in the potential benefit of segmentation.

The liquid metal coils feature reliable sealing and experience no chemical deterioration such as oxidation and corrosion. They readily integrate into a textile, which was chosen to provide suitable mechanical properties for stretching and release in concert with the conductors while being lightweight and comfortable for patients to wear. The proposed detector garments are unobtrusive and adapt automatically to body parts to be imaged, improving sensitivity and spatial encoding performance in parallel imaging. Towards prospective routine use, the electrical and mechanical considerations reported in this work will need to be complemented by design efforts with a focus on ergonomics, workflow, and hygiene. Solutions in these respects are expected to benefit greatly from ongoing advances in textile technology.

Wearable MR coil arrays expand the scope of magnetic resonance imaging. Static angle imaging and kinematic studies can provide functional insights accessible at high sensitivity with a wearable array. Kinematic imaging equips MRI with functionality so far reserved to fluoroscopy in X-ray imaging and ultrasound. MRI, however, provides this imaging functionality without associated risks of harmful radiation and no need for an acoustic window.

Resembling ordinary garments, wearable coil arrays can be put on by patients themselves, potentially saving setup time at the MR system. Overcoming the previous tradeoff between sensitivity and patient comfort, wearable arrays mark a shift towards MR technology that adapts to the patient rather than the other way around.

In light of the promise of a more patient-centered way of MRI, rethinking the interface to the MR scanner also becomes more urgent. Cabling from the coil array to the MR acquisition system can pose safety concerns for patients upon coupling to the RF transmitter and they complicate coil array handling especially for high channel counts. Elimination of galvanic connections may be realized through optical or ultimately wireless signal transmission. A harsh electromagnetic environment renders this task difficult but in-bore reception has been achieved with discrete electronics^27^ and on-chip^28^. Large amounts of data are transmitted via optical fibers. A wireless link for MR data transmission^29^ has been proposed and enabling technologies such as wireless power transfer^30^ and wireless detuning of receive coils^31^ are currently under development.

## Methods

### Liquid metal coil construction

Eutectic Gallium Indium (eGaIn) liquid metal (Sigma Aldrich, Buchs, Switzerland) with a melting point of 15.7 °C was used as conductor material. The alloy is composed of 75.5% Gallium and 24.5% Indium by weight. Its density is 6.25 g/cm^3^ at 25 °C. The material’s resistivity^25^ is $29.4\,\cdot \,{10}^{-8}\,\Omega m$.

Vinyl-Methyl-Silicone tubes (Laborshop24 GmbH, Gross-Zimmern, Germany) with a hardness of 55–60 Shore A (DIN 53505) were used. The material’s density is 1.14 g/cm^3^. The tubes’ temperature resistance is specified to be −60 °C to +200 °C and elongation at break to be more than 500%. For liquid metal coil building, silicone tubes with inner diameters of 0.3, 0.5, 0.8 and 1.0 mm and corresponding outer diameters of 0.7, 1.3, 1.6 and 1.8 mm were used.

Coils of all inner diameters for use in single coil quality factor measurements, SNR evaluation and multi channel SNR evaluation were contacted using copper litz wire (LIFY 0.25 mm^2^ copper, Kabeltronik, Denkendorf, Germany). For the wearable coil array, 0.8 mm coils were contacted using a custom made copper insert produced on a turning machine for enhanced sealing performance. The insert has a total length of 17 mm, consisting of a 15 mm body with a diameter of 1.3 mm and a 2 mm extension with a diameter of 0.6 mm. For sealing of the contact, Cyanacrylat glue on Methoxylethyl basis (MD Glue Xtreme 3, Suter Kunststoffe AG, Fraubrunnen, Switzerland) and heat shrink tubing (CGPT-R-2.4-0, 2:1, 2.4 mm, RAYCHEM-TE Connectivity, Schaffhausen, Switzerland) were used.

For fabrication of the liquid metal coils, the silicone tube was cut to length. A syringe with needle diameter matching the tube inner diameter was used to avoid air enclosures. The needle was inserted into the silicone tube by about 10 mm and the liquid metal was injected. The copper litz wire or custom insert was inserted first on one side, which removed the remaining air in the tube, followed by the other side. Instant adhesive and heat shrink tubing were applied. The coils were let dry for several hours or overnight before they were subjected to any mechanical stress.

### Single coil bench measurements

A custom made decoupled loop probe connected to a network analyzer (E5061B vector network analyzer, Agilent Technologies, Santa Clara, USA) was used for coil characterization. The decoupled loop probe was decoupled in free space to about −80 dB. Unloaded and loaded quality factors were measured using the network analyzer’s integrated Q measurement function. Unloaded Q was evaluated for coils with tube inner diameters of 0.3, 0.5, 0.8 and 1.0 mm. All coils were resonated using a 2.7 pF capacitor (American Technical Ceramics, Huntington Station, USA) and radially stretched from 0 to 60% in 10% steps. To apply this strain, a laser cut setup consisting of 6 discs made of PMMA was used. The discs were 10 mm thick and had increasing diameters ranging from 105 mm to 168 mm in 10% steps. A resonance frequency shift occurs since coils are resonated with a capacitor of fixed value and the inductance of the coil changes with strain. Unloaded Q values were measured at these respective resonance frequencies. To assess how the unloaded Q factor changes over time, the Q values were rechecked after 7 weeks.

Breakdown strain of the silicone tube and contact were tested. Five segments of 0.8 mm inner diameter silicone tube and of 20 cm length including a loop for attachment were used. Loops were connected to a 5 kg tension spring balance, while the open end was fixated on a measurement setup using a screw clamp. Measurement tape extended from the screw clamp towards the strain direction and enabled readout of elongation of the silicone tube at breakdown relative to the relaxed state. To assess breakdown strain for contacts, three segments of 0.8 mm inner diameter silicone tube at a length of 8 cm including a loop were attached to custom copper inserts using instant adhesive and heat shrink tubing. The copper insert was attached to the measurement setup through a screw clamp and the force necessary for contact failure was measured using a 5 kg tension spring balance connected to the loop.

Cyclic strain testing was performed with a 10 cm diameter liquid metal coil manufactured from 0.8 mm inner diameter silicone tube. The coil was resonated with a 2.7 pF capacitor (American Technical Ceramics, Huntington Station, USA) mounted on a small custom printed circuit board. This coil was then attached to common athletic pants fabric by means of 16 rubber band loops at 8 equidistant positions. On two opposite sides of the fabric a hem was machine-sewed into which two bars were inserted. These bars were inserted into indentations on a laser cut PMMA experiment setup that allows for precise strain in one direction. The relaxed coil formed a circle with diameter 100 mm and an ellipse with a long axis of 135 mm and a short axis of 85 mm when strained. Strain was applied 950 times with the contact of the coil perpendicular to the strain axis and 950 times with the contact in line with the strain axis. Unloaded Q was measured with a decoupled loop probe connected to a network analyzer every 50 strain repetitions.

### Single coil SNR measurements

Four liquid metal coils with a diameter of 105 mm were fabricated with silicone tubes of inner diameters of 0.3, 0.5, 0.8 and 1.0 mm. Their Q_unloaded_/Q_loaded_ was 61/17, 110/16, 152/16 and 166/16, respectively. A reference coil of the same diameter of 105 mm was fabricated from 0.8 mm diameter copper wire. The Q_unloaded_/Q_loaded_ of this copper reference coil was 278/18. All coils were connected to a custom printed circuit board containing tuning, matching, and detuning electronics together with an integrated preamplifier module, with properties similar to standard metal-oxide-semiconductor field-effect transistors^7^. Printed circuit boards and components were individual for each coil. The same preamplifier was used in all coils to reduce the effects on the measurements due to variation of noise figure over preamplifiers. Images were acquired on a Philips 3T Ingenia MR system (Philips Healthcare, Best, The Netherlands) using a gradient echo sequence (TR 30 ms, TE 4.7 ms, FA 30°, 1 × 1 × 5 mm^3^, 1 slice, scan duration 6 s) with the coils conforming to a 120 mm diameter phantom bottle containing a copper sulfate solution emulating MR characteristics of human tissue (770 mg/l CuSO_4_ ∙ 5(H_2_O), 2000 mg/l NaCl). Following the method proposed by Nordmeyer-Massner *et al.*,^7^ pixelwise SNR maps were calculated from imaging data and a dynamic noise scan with no RF and no gradients.

### Cylindrical receive array construction

Four 13.5 cm diameter liquid metal coils, segmented twice, were made from 0.8 mm inner diameter silicone tube. The coils were resonated with 3.3 pF capacitors (American Technical Ceramics, Huntington Station, USA) and provided a Q_unloaded_/Q_loaded_ of 146/15, 165/16, 155/16 and 158/17. Following the approach by Nordmeyer-Massner *et al*.^7^, each coil was interfaced to a high-Z preamplifier through a π-matching network. The π-matching network, together with the coil, provided a characteristic double-peak frequency response^7^ and the preamplifier was selected because it exhibits only moderate noise figure degradation upon deviation from its optimum source impedance^20^. This combination provided robustness to frequency shifts and impedance mismatch, which occur due to variable loading and stretching of coils. Coils were sewn onto a common knee bandage (Bort GmbH, Weinstadt-Benzach, Germany) using a zigzag stitch. They were overlapped for approximate geometric decoupling, which was complemented by preamplifier decoupling.

### Phantom imaging

Three different sized phantoms (770 mg/l CuSO_4_ ∙ 5(H_2_O), 2000 mg/l NaCl) of diameters of 120 mm, 130 mm and 140 mm were used for SNR evaluation of the cylindrical array when stretched. Tuning and matching of the receive array was performed only when attached to the phantom of 120 mm diameter. For the two larger phantoms no further adjustments to tuning and matching were made. Images were acquired on a Philips 3T Ingenia MR system (Philips Healthcare, Best, The Netherlands) using a gradient echo sequence (TR 30 ms, TE 4.7 ms, FA 30°, 1 × 1 × 5 mm^3^, 1 slice, scan duration 6 s). From images and noise data from a dynamic noise scan, pixelwise SNR and noise correlation maps were calculated^7^.

### Wearable knee coil array construction

The coil array outline was laser cut from PMMA and transferred for sewing to the upper one of two layers of a stretchable textile taken from common athletic pants (H&M, Stockholm, Sweden). Joining the two layers of textile by machine sewing, formed textile ducts into which the individual coil elements were inserted. Four liquid metal coils made from 0.8 mm inner diameter silicone tube were fabricated for the wearable array. The anterior part of the array consisted of three coils, segmented twice, sized 8 cm × 16 cm with Q_unloaded_/Q_loaded_ of 190/13, 180/13 and 180/13. The posterior part of the array was formed by one large trapezoidal coil with five-fold segmentation. It was sized 24 cm (top) ×17 cm (sides) ×16 cm (bottom) and yielded a Q_unloaded_/Q_loaded_ of 155/14. All coils were connected to custom textile-embedded electronics. A printed circuit board with a size of 20 mm × 26.5 mm was designed using printed circuit board design software (Altium Designer, Altium Ltd., Chatswood, Australia). Tuning, active and passive detuning and π-matching circuitry employed non-magnetic SMD capacitors (American Technical Ceramics, Huntington Station, USA) and inductors (Coilcraft Inc., Cary, USA). An integrated preamplifier module, with properties similar to standard metal-oxide-semiconductor field-effect transistors^7^ was used. The preamplifier’s input impedance is 2 kΩ at 127.8 MHz^7^ and its noise figure is 0.83 dB for optimal source impedance^20^. The preamplifier was selected because it demonstrates only moderate noise figure degradation for deviations from its optimum source impedance^20^. Combined with the π-matching network, it provided robustness to frequency shifts and impedance mismatch, which occur due to variable loading and stretching of coils. A fuse (Littelfuse Inc., Chicago, USA) was included in series with the coil conductor. Foam tape was applied on the side of the printed circuit board facing the inside of the wearable coil array. This ensured a distance of several millimeters between coil electronics and the volunteer, a standard precaution against RF currents, heating and mechanical injuries.

### *In vivo* imaging

All images were acquired on a 3T Philips Ingenia MR system (Philips Healthcare, Best, The Netherlands). Magnitude images were reconstructed on the scanner console employing sensitivity correction. SNR maps were calculated from complex data using Matlab (The MathWorks Inc., Natick, USA).

Tuning and matching of the wearable receive coil array was performed only for the straight position of the knee. For imaging at different flexion angles and kinematic imaging, no further adjustments were made to the coil array. Imaging of a healthy volunteer’s knee at three different flexion angles was performed using a RARE sequence (TR 523 ms, TE 9 ms, 0.6 ×0.69 ×3 mm^3^, 17 slices, scan duration 3:30 min).

For comparison of the 4-channel wearable coil array to an 8-channel commercial coil array, imaging was performed using a gradient echo sequence (TR 500 ms, TE 5.8 ms, FA 30°, 0.7 ×0.7 ×3 mm^3^, 24 slices, 2 dynamics, scan duration 8:55 min). Pixelwise SNR maps were calculated^7^ from images and noise data from a dynamic noise scan with no RF and no gradients.

A MR compatible knee support was designed using computer aided design software (NX, Siemens Industry Software Inc., Plano, USA) and manufactured from PMMA. This knee support (Supplementary Fig. S3) was used for static angle imaging shown in the comparison of the wearable coil array to the commercial coil array and for kinematic imaging.

Kinematic imaging (Supplementary Vid. S1) was performed using a SPGR sequence (TR 9.9 ms, TE 5.9 ms, FA 15°, 1 ×1 ×5 mm^3^, 1 slice, 30 dynamics, total scan duration 1:22 min). Supplementary Video S1 was generated from 30 acquired dynamics measured during one flexion of a volunteer’s knee joint from straight to flexed position.

Safety of the liquid metal coils was assessed with heating measurements during phantom imaging employing a sequence with high specific absorption rate. RF transparency of the coil array was assessed by comparison of body coil phantom images with and without the coil array present in the scanner. RF safety during *in vivo* scans was ensured by single coil data reconstruction to verify proper functionality of all coil elements and by inspection of a body coil image to check receive coil array transparency.

The study was performed with ethical approval from the *Cantonal Ethics Committee Zurich* in Zurich, Switzerland and in accordance with all applicable regulations. Informed consent was obtained from the volunteer.

## Supplementary information

Supplemental information.

Supplementy Video S1.

## Data Availability

Supporting data are available from the corresponding author upon reasonable request.
