# Publisher Correction: Detector clothes for MRI: A wearable array receiver based on liquid metal in elastic tubes

**DOI:** 10.1038/s41598-020-69151-3

**Published:** 2020-07-17

**Authors:** Andreas Port, Roger Luechinger, Loris Albisetti, Matija Varga, Josip Marjanovic, Jonas Reber, David Otto Brunner, Klaas Paul Pruessmann

**Affiliations:** 1 Institute for Biomedical Engineering, ETH Zurich and University of Zurich, Zurich, Switzerland; 2 Institute for Electronics, ETH Zurich, Zurich, Switzerland

Correction to: *Scientific Reports* https://doi.org/10.1038/s41598-020-65634-5, published online 01 June 2020

A supplementary file containing Supplementary Video S1 was omitted from the original version of this Article. This has been corrected in the HTML version of the Article; the PDF version was correct at time of publication.

