# Supplementary material for: Detector clothes for MRI: A wearable array receiver based on liquid metal in elastic tubes
Source: Sci Rep. 2020 Jun 1;10:8844. doi: 10.1038/s41598-020-65634-5 (PMC7264329; doi:10.1038/s41598-020-65634-5)
Supplement: Supplementary file 1 — Supplemental information. [file 41598_2020_65634_MOESM1_ESM.pdf]

Supplementary Material

Detector clothes for MRI: A wearable array receiver based on liquid metal in elastic tubes

Andreas Port[1], Roger Luechinger[1], Loris Albisetti[1], Matija Varga[2], Josip Marjanovic[1], Jonas Reber[1], David Otto Brunner[1], and Klaas Paul Pruessmann[1*]
[1]Institute for Biomedical Engineering, ETH Zurich and University of Zurich, Zurich, Switzerland
[2]Institute for Electronics, ETH Zurich, Zurich, Switzerland

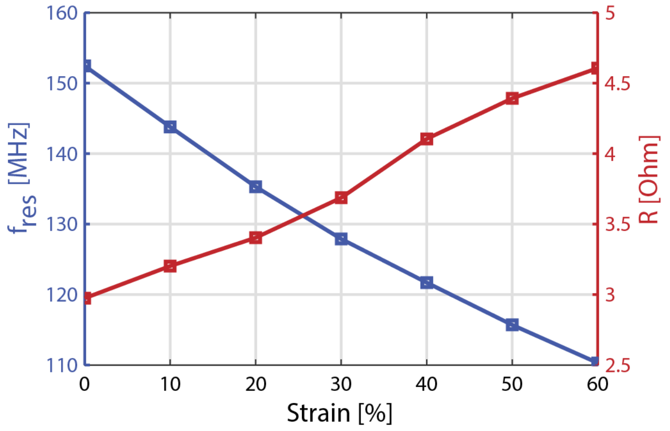

Supplementary Figure S1 | Resonance frequency and resistance of 0.8 mm inner diameter tube coil under strain. Resonance frequency f_{res} and resistance R of the 0.8 mm inner diameter tube coil are shown for strains of 0 to 60%.

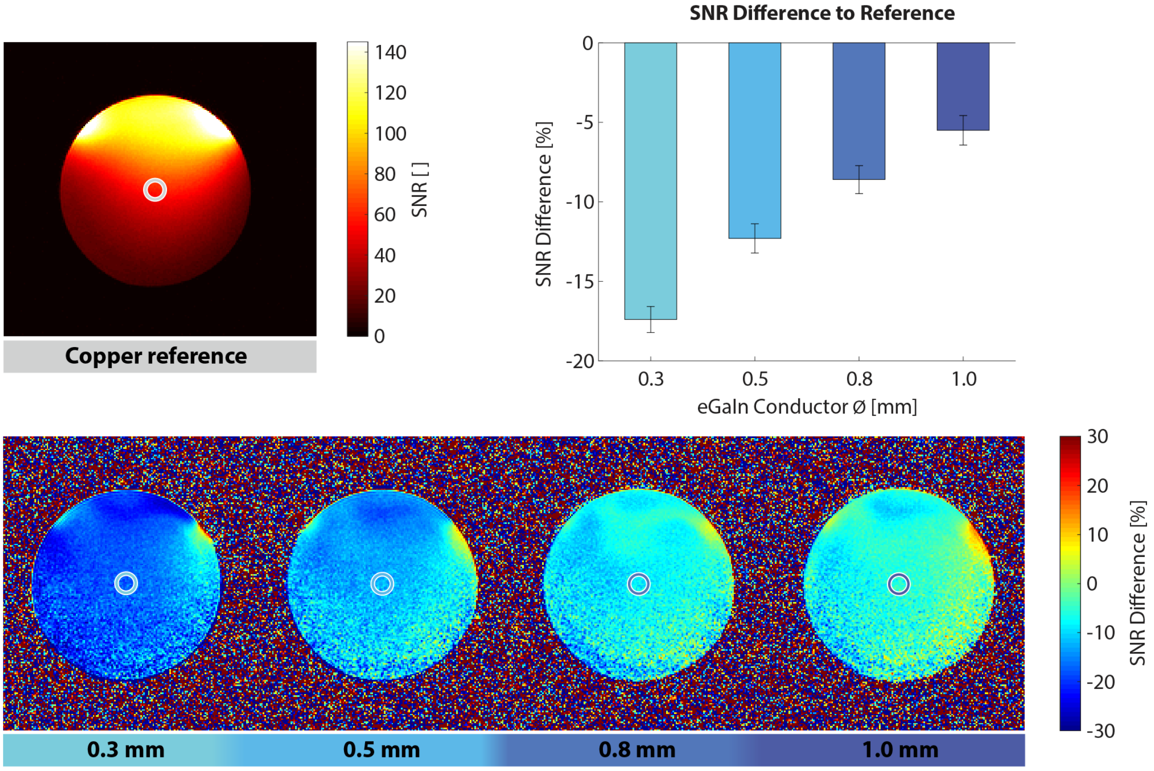

Supplementary Figure S2 | SNR difference comparison of liquid metal coils to copper reference coil.
SNR was measured in a transverse plane through a phantom emulating MR characteristics of human tissue. Difference SNR maps to a copper reference coil were calculated for liquid metal coils with tube inner diameters of 0.3, 0.5, 0.8 and 1.0 mm. SNR difference values larger than 30 % or smaller than -30 % were set to 30 % and -30 %, respectively. Slight differences in the positioning of coil conductors result in SNR difference variations at the location of coil conductors. SNR differences are plotted as mean SNR difference inside a circular region of interest in the center of the phantom. Error bars show standard deviation of the SNR difference data.

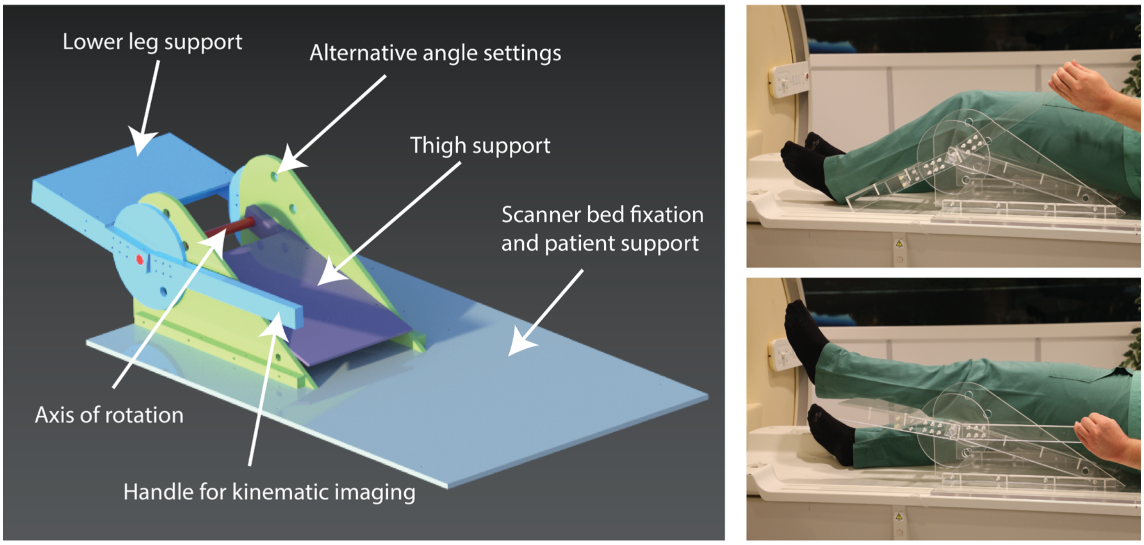

Supplementary Figure S3 | A MR compatible knee support for static and kinematic *in vivo* imaging.
The knee support is fixed on the MR scanner bed and stabilized through the volunteer's body weight. It provides thigh and lower leg support. The knee joint rests above the axis of rotation. Various angles can be set for static imaging. A handle allows for stepless kinematic imaging.

Supplementary Video S1 | Kinematic *in vivo* knee imaging.
Movie generated from 30 dynamics of a sagittal slice through the volunteer's knee acquired with a SPGR sequence. The knee is flexed continuously during the scan from straight to bent position.
